# Precise Photodynamic Therapy of Cancer via Subcellular Dynamic Tracing of Dual-loaded Upconversion Nanophotosensitizers

**DOI:** 10.1038/srep45633

**Published:** 2017-03-31

**Authors:** Yulei Chang, Xiaodan Li, Li Zhang, Lu Xia, Xiaomin Liu, Cuixia Li, Youlin Zhang, Langping Tu, Bin Xue, Huiying Zhao, Hong Zhang, Xianggui Kong

**Affiliations:** 1 State Key Laboratory of Luminescence and Applications, Changchun Institute of Optics, Fine Mechanics and Physics, Chinese Academy of Sciences, Changchun, Jilin, China; 2 The First Hospital of Jilin University, Changchun 130021, China; 3 Van’t Hoff Institute for Molecular Sciences, University of Amsterdam Science Park 904, 1098 XH Amsterdam, The Netherlands

## Abstract

Recent advances in upconversion nanophotosensitizers (UCNPs-PS) excited by near-infrared (NIR) light have led to substantial progress in improving photodynamic therapy (PDT) of cancer. For a successful PDT, subcellular organelles are promising therapeutic targets for reaching a satisfactory efficacy. It is of vital importance for these nanophotosensitizers to reach specifically the organelles and to perform PDT with precise time control. To do so, we have in this work traced the dynamic subcellular distribution, especially in organelles such as lysosomes and mitochondria, of the poly(allylamine)-modified and dual-loaded nanophotosensitizers. The apoptosis of the cancer cells induced by PDT with the dependence of the distribution status of the nanophotosensitizers in organelles was obtained, which has provided an in-depth picture of intracellular trafficking of organelle-targeted nanophotosensitizers. Our results shall facilitate the improvement of nanotechnology assisted photodynamic therapy of cancers.

Photodynamic therapy (PDT), involving molecular oxygen, photosensitizer and excitation light, is a noninvasive treatment modality in clinic for a variety of diseases^1^,^2^,^3^,^4^. Restriction of this modality comes from the low tissue penetration depth of excitation light (ultraviolet or visible), the poor accumulation of photosensitizers on tumor sites, especially in subcellular organelles or structures and the difficulty in performing precise treatment^5^,^6^,^7^,^8^,^9^. In recent years, nanotechnology has been merged with proof of concept that it may circumvent these problems. Typically lanthanide-doped upconversion nanoparticles (UCNPs) have been regarded as promising candidates for near-infrared (NIR) light-triggered deep tumor PDT. UCNPs are capable to emit various colors of light upon irradiation of continuous-wave NIR laser. Numerous reports have demonstrated that most of the current clinical photosensitizers uploaded on surface of UCNPs can be activated with NIR, thus applying PDT on lesions in deep tissue is becoming feasible^10^,^11^,^12^,^13^,^14^. However, the requirements of the relatively high irradiation power and long exposure time of NIR can easily lead to a biological damage^10^,^15^. On the other hand, subcellular organelles are promising therapeutic targets for effective PDT, where the precisely triggering time and targeting locations are crucial. It should be noted that the surface properties of UCNPs are related to the cellular uptake, the dynamics of intracellular delivery and the targeting of UCNPs-PS. Till now, however, intracellular delivery of UCNPs-PS remains unclear. Moreover, it is known that the lifetime of singlet oxygen is very short (<40 ns) in biological systems, which leads to a very limited diffuse distance (radius of action: <20 nm)^16^,^17^. Accordingly, the extent of phototoxicity is highly dependent on the intracellular accumulation level, subcellular localization of UCNPs-PS and trigger time. Therefore, a distinct picture of the migration and localization dynamics of the UCNPs-PS in cancer cells and their relation to PDT are the prerequisite in determining the starting- and duration time of the therapy.

To study the dynamics of the migration and localization of the UCNPs-PS in subcellular organelles and their relation with PDT, the UCNPs-PS must be optimized with a maximal energy transfer from UCNPs to PS which is closely interrelated with the yield of singlet oxygen (^1^O_2_)^11^,^18^. It is known that the energy transfer is affected by the upconversion luminescence efficiency of the donor, the spectral overlap between the donor emission and photosensitizer (acceptor) absorption, the distance between the two, and the amount of photosensitizing molecules loaded on each UCNP. In recent years our group has proposed and pioneered the method of covalent conjugation of photosensitizer to UCNP, which is able to effectively eliminate the leakage of photosensitizers from UCNPs, shorten the energy transfer distance and ensure a high energy transfer efficiency^19^. On top of that, we have demonstrated that there exists an optimal shell thickness for the highest production of ^1^O_2_ for UCNPs-PS^20^,^21^. Recently cytotoxicity and PDT effect of UCNPs-PS have drawn the attention of the researchers, but few reports on the cellular uptake, dynamic process of intracellular delivery of UCNPs-PS^22^, although these play also a crucial role for a successful PDT.

In this work, the subcellular migration dynamics of the nanophotosensitizers, the corresponding subcellular mechanism of PDT were studied for the first time (Fig. 1), employing the as-prepared core/shell UCNPs-PS modified with poly(allylamine) (PAAm) and dual-loaded with the Rose Bengal (RB) and Zinc(II) phthalocyanine. Our study indicates that the PAAm-UCNPs-PS can be well taken up by cells and migrate from endosomes/lysosomes to cytoplasm and then to mitochondria in cells. The most effective PDT was obtained when plenty of the nanophotosensitizers accumulate in mitochondria. The cell death mechanism was further proved to be the mitochondria-related aoptosis pathway.

## Results and Discussion

### Preparation of amino-functionalized core/shell UCNPs with RB and ZnPc photosensitizers (UCNPs-RB&ZnPc)

**Figure 2:**
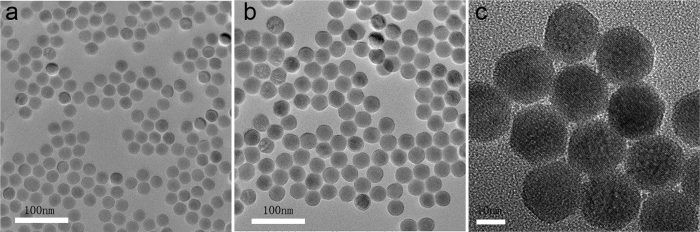
TEM images of (**a**) NaYF_4_:Yb^3+^,Er^3+^ UCNPs, scale bar, 100 nm; (**b**) NaYF_4_:Yb^3+^,Er^3+^@NaYF_4_ core/shell UCNPs, scale bar, 100 nm; (**c**) the functionalized UCNPs nanoplatform with RB and ZnPc photosensitizers. Scale bar, 10 nm.

To obtain prominent visible emission at 525 nm, 540 nm and 650 nm, and the optimal energy transferred from the UCNPs to photosensitizers, core/shell structure, *i.e.* NaYF_4_: 20%Yb^3+^, 2% Er^3+^@NaYF_4_ UCNPs, was adopted. The size distribution and morphology of the UCNPs are assured in Fig. 2. It could be seen that the hexagonal core and the core/shell UCNPs consist of uniform and nearly monodispersed nanoparticles of 24 ± 1.0 nm and 32 ± 0.9 nm in diameter, respectively. Compared with the core (NaYF_4_: 20%Yb^3+^,2% Er^3+^), the mean size of core/shell UCNPs increased by ∼8 nm, indicating the formation of ~4 nm shell, which is the best for transferring the emission energy of UCNPs to the surface bonded photosensitizers^20^,^21^. Subsequently, the two photosensitizers RB and ZnPc were anchored on the surface of UCNPs following the co-loading strategy to allow a condensation reaction between the amino group of the UCNPs and the carboxyl group of the RB and ZnPc to form amide bonds, which is more beneficial to higher ^1^O_2_ yield, compared with the silica adsorption method of Zhang *et al*.^23^.

**Figure 3:**
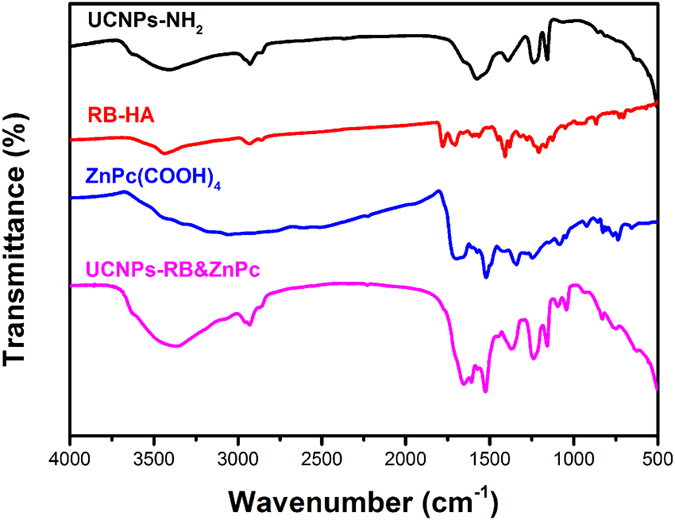
FT-IR spectra of amino-functionalized NaYF4:Yb^3+^, Er^3+^@NaYF_4_ UCNPs (black curve), RB-HA (red curve), ZnPc(COOH)4 (blue curve) and UCNPs-RB&ZnPc nanoplatform (pink curve).

In their approach, the photosensitizers were imbibed within a thick mesoporous silica shell through physical adsorption, which enlarged the energy transfer distance and inevitably suffered from untimely release of photosensitizer from UCNPs during blood circulation. In Fig. 3, the absorption peak at 1576 cm^−1^ in the FT-IR spectrum of UCNPs with amino ligands indicates the N-H deformation vibration. For a free photosensitizer, the absorption peak at 1710 cm^−1^ is associated with the conjugated C = O stretching vibration mode of the carboxyl group. After conjugating with NH_2_-UCNPs, the relevant absorption of -COOH and -NH_2_ disappears and two new peaks appear at 1654 cm^−1^ and 1525 cm^−1^, corresponding respectively to the C=O stretching vibration and N-H bending vibration modes of secondary amide. The UCNPs-PS was constructed considering selective energy transfer between the excited states of UCNPs and the matched ground states of photosensitizers.

**Figure 4:**
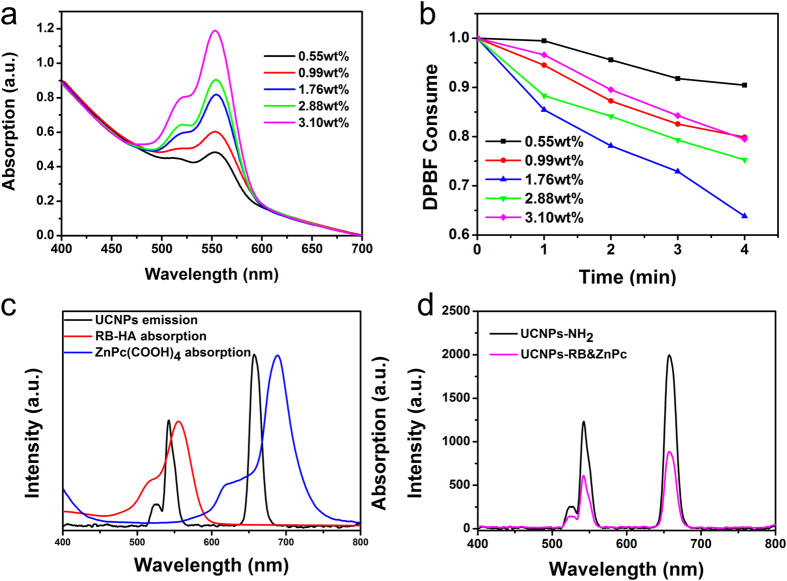
(**a**) UV-Vis absorption spectra of UCNPs nanoplatform covalently bonded with RB-HA of different concentrations. The weight percentage of RB-HA per UCNPs nanoconjugates is also given. (**b**) Consumption of DPBF over time with 980 nm irradiation due to ^1^O_2_ generation from different weight percentage of RB-HA per UCNPs nanoconjugates constructed in the covalent way. (**c**) Upconversion luminescnece spectrum of UCNPs (black curve) as donor and the absorption spectrum of the RB-HA (red curve) and ZnPc(COOH)_4_ (blue curve) as acceptors. (**d**) Upconversion luminescence spectra of UCNPs (black curve), UCNPs-RB&ZnPc conjugates (pink curve) under optimum co-loading condition with 980 nm light excitation.

As evidenced in Fig. 4c, the absorption spectra of RB and ZnPc overlaped very well with the upconversion luminescence green band (520–570 nm) and red band (630–650 nm), respectively. The yield of singlet oxygen generation decreased with the increase of photosensitizer’s concentration due to the concentration quenching effect^24^. To reach maximal singlet oxygen yield, the optimal loading amount of ZnPc was set based on our previous work (6% w/w)^25^, and the optimal RB loading amount was obtained by monitoring the ^1^O_2_ production. The RB absorption increased with the increase of of the number of the conjugated RB molecules (Fig. 4a), and the corresponding singlet oxygen production was probed by chemical probe DPBF (Fig. 4b), from which the optimal loading amount of RB was determined to be 1.76% (w/w). Under excitation at 980 nm, the green and the red emitting bands of UCNPs were quenched by RB and ZnPc, respectively, indicating the presence of energy transfer between them. The energy transfer efficiency was estimated from the quenching of the UCL emission intensity: E = (I_0_ − I_1_)/I_0_, where I_0_ and I_1_ are emission intensities of UCNPs before and after conjugation with photosensitizer, respectively. From this formula, the energy transfer efficiency under optimal co-loading condition was determined to be 49.1% for the UCL band at 540 nm, and 54.2% for the UCL band at 650 nm (Fig. 4d).

### Singlet oxygen generation of UCNPs-RB&ZnPc

**Figure 5:**
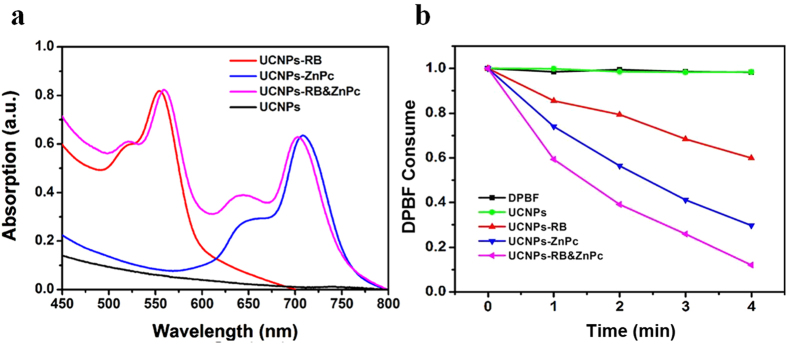
(**a**) UV-Vis absorption spectra of UCNPs, UCNPs-RB, UCNPs-ZnPc, UCNPs-RB&ZnPc. (**b**) Consumption of DPBF over time due to ^1^O_2_ generation for DPBF only, UCNPs, UCNPs-RB, UCNPs-ZnPc, UCNPs-RB&ZnPc with the same power density 980 nm irradiation.

To verify the superiority of dual-loaded strategy, we have compared the singlet oxygen production of the UCNP-RB&ZnPc, UCNPs-RB and UCNPs-ZnPc. From the absorption spectra, the loading amounts of ZnPc (650 nm) and RB (540 nm) of co-loading sample UCNP-RB&ZnPc were determined to be 5.9% and 1.77%, respectively, similar to the optimized single loading ones (6% for ZnPc and 1.76% for RB) (Fig. 5a). In this case, singlet oxygen production was measured as shown in Fig. 5b, and the relationship between DPBF consumption and the irradiation time was thus built up. The control experimental results are also shown, where the upper two lines (green and black) are for DPBF incubated with nano-conjugates without and with 980 nm light irradiation, respectively. The results illustrate that singlet oxygen can be significantly enriched by co-loading. After 4 min irradiation nearly 90% of the DPBF was consumed for co-loading sample. The high efficiency of ^1^O_2_ generation arises from the optimal ratio of ZnPc and RB loading, as well as the optimal shell thickness for energy transfer between UCNPs and PS.

### Cellular uptake distribution of UCNPs-RB&ZnPc

**Figure 6:**
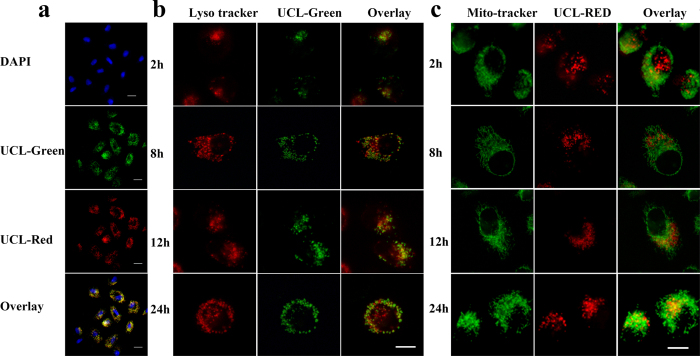
(**a**) Assessment of UCNPs-PS cellular uptake via confocal laser-scanning microscopy using lung carcinoma A549 cells. (**b**) The cellular internalization and subcellular distribution of nanoplatform in lysosome (2 h, 8 h, 12 h and 24 h) and (**c**) mitochondrial (2 h, 8 h, 12 h and 24 h). Cells were stained with LysoTracker Red and MitoTracker Green, respectively. Scale bar, 10 μm.

The cellular uptake of UCNPs-RB&ZnPc, time dependent PDT and corresponding mechanisms were studied. The delivery efficiency of the nanoplatforms on A549 cells was examined by modified confocal laser scanning microscope (CLSM). In Fig. 6 the blue emission came from DAPI, which stained cell nucleus and the green and the red emissions were from the UCL at 540 nm and 650 nm, respectively. It can been seen that the UCNPs-RB&ZnPc were internalized inside the cells (Fig. 6a, top), illustrating the endocytic uptake of UCNPs-RB&ZnPc. To further study intracellular transport of the nanoplatforms two main organelles (lysosome and mitochondria) were stained. In Fig. 6b,c lysosomes were stained in red and UCNPs-PS in green, or mitochondria were marked in green and UCNPs-PS in red. The co-localization of UCNPs-PS in lysosome and mitochondria produced a yellow fluorescence in the merged images. Understanding the intracellular localization within the endolysosomal compartments and the fate of nanoparticles with respect to their uptake profile is crucial in designing a nanoplatform and performing a precise and efficient PDT.

Comparison between the distributions of UCNPs-PS in mitochondria and lysosome at different times (Fig. 6b) turned out that the nanoplatform appeared in the lysosomes after 2 h incubation, and accumulated more and more after 8 h, 12 h and 24 h. Interestingly, the nanoplatforms gradually translocated to mitochondria after 12 h incubation and accumulated there more after 24 h incubation (Fig. 6c). The spatial co-localization of UCNPs-PS with mitochondria was further confirmed by confocal Z-scan UCL imaging in Figure S2 (supporting information). It can thus be concluded that UCNPs-PS, once entering the cell, will firstly accumulate in endosome/lysosomes and migrate from endosome/lysosome to the cytoplasm and then to the mitochondria. The escape of nanoplatform might come from the remaining surface ligand of NH_2_-PAAm, which has the ability to accept the protons, leading to the osmotic pressure buildup across the organelle membrane. The osmotic pressure might cause disruption of the acidic endosomes and release of the trapped materials into the cytoplasm^26^. The translocation of nanoplatforms makes the integrity of the lysosomal membrane of A549 cells impaired, as probed by Acridine orange (AO) (Figure S1). AO is a lysomotropic metachromatic fluorescent dye, exhibits red fluorescence as protonatedoligomeric form in complete lysosome and shows green fluorescence as monomer deprotonated form in the cytoplasm. After the uptake and redistribution of the nanoplatforms, red florescent decreased and in the meantime green florescence increased, confirming the endosomal/lysosomal escape of nanopatform^27^,^28^,^29^. The targeting delivery and gradual accumulation in mitochondria may be attributed to the positively charged UCNPs-RB&ZnPc nanoplatforms (zeta potential = 31.4 mV) and surface anchored lipophilic PS molecules. The accumulation in organelles of mitochondria might promote apoptosis after PDT.

### *In vitro* PDT and Cell Death Pathways

**Figure 7:**
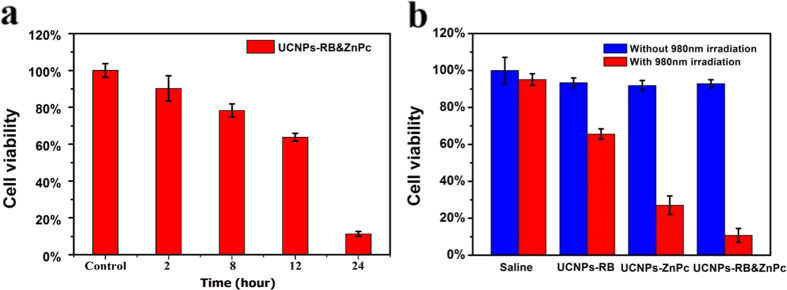
(**a**) Viability of cells treated with UCNPs-RB&ZnPc at concentration of 100 μg/mL under 980 nm laser excitation with density of 0.25 W/cm^2^ at different times (2 h, 8 h, 12 h and 24 h). Error bars are based on the results of six samples. (**b**) Viability of cells treated with saline, UCNPs, UCNPs-RB, UCNPs-ZnPc, UCNPs-RB&ZnPc with (red) or without (blue) 0.25 W/cm^2^ laser irradiation at 980 nm. Concentrations are 100 μg/ml. Standard deviations are shown (n = 4).

**Figure 8:**
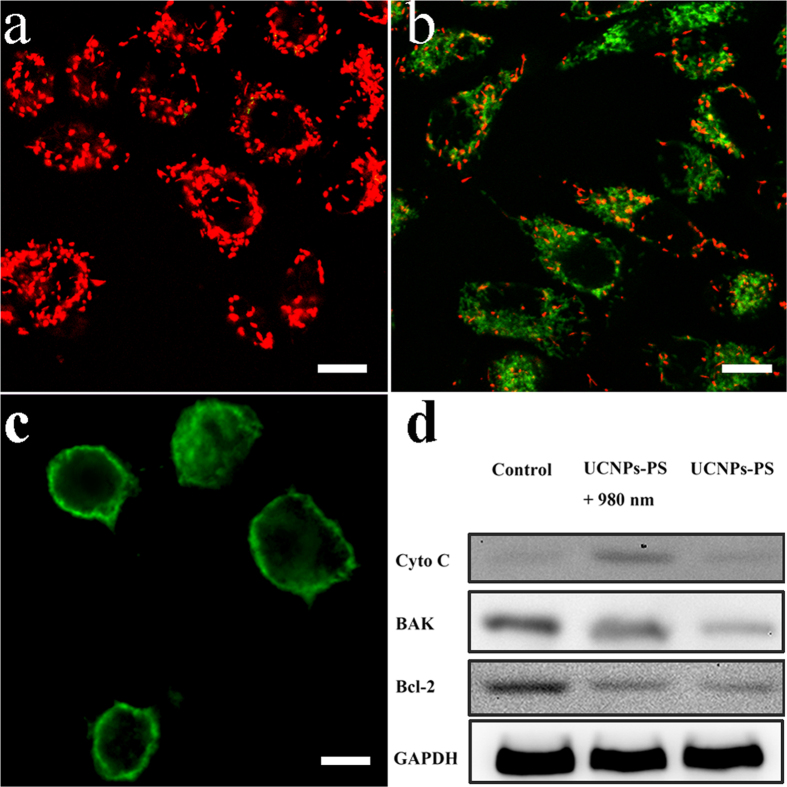
NIR-triggered PDT induced mitochonodrial damage in A549 cells. Cells treated with UCNPs-RB&ZnPc (100 μg/mL) were irradiated with a 980 nm laser and stained with JC-1. (**a**) The mitochondrial membrane potential of A549 cells without PDT (**b**) Increase of green fluorescence in A549 cells with PDT, suggesting the loss of mitochondrial membrane potential. (**c**) NIR-triggered PDT induced apoptosis and necrosis of A549 cells with 100 μg/mL of UCNPs-RB&ZnPc and stained with annexin V-FITC and PI. The cell membranes were stained only in green indicating the early apoptotic stage. (**d**) Expression of Bcl-2 family proteins (i.e., Bak and Bcl-2) and Cyto C protein by Western blotting analysis after PDT, GAPDH was used as an internal control. PDT condition: 0.25 W/cm^2^, 15 min, 3 min break after 5 min of irradiation. Scale bar, 10 μm. Uncropped Western blot gels related to this figure are displayed in Suppl. Figure S5.

Now we turn to the effect of the nanoplatform migration on PDT assisted by MTT method (Fig. 7a) with 980 nm light excitation and dosage of 100 μg/mL. Most of the nanoplatforms accumulated in mitochondria after 24 h incubation and the lowest cell survival rate was 20% after PDT. When the nanoplatforms were partially enriched in mitochondria after 12 h incubation, the PDT led to a survival rate of about 70%. On the contrary, the PDT effect was poor when the nanoplatforms were enriched in lysosome only before 12 h. Bearing these in mind, we carried out the following effective and precise PDT and also compared the PDT effects between co-loaded UCNPs-RB&ZnPc and singly loaded counterparts. To do so, cancer cells were incubated with co-loading and single-loading UCNPs-PS, respectively. The cell viability was determined from a standard MTT assay. Figure 7b provides the PDT effect on A549 cells with dosage of 100 μg/mL. No significant decrease in cell viability was observed in the control group without 980 nm irradiation. In contrast, the same power density of 980 nm light had far more obvious killing effect for dual-loaded UCNPs-RB&ZnPc nanoplatforms, compared with those loaded with only one kind of photosensitizers. As is known, the mitochondria not only provide energy from ATP by the process of oxidative phosphorylation, but also decisively regulate the intrinsic pathway of apoptosis, which is regarded as the major mode of cell death in cancer therapy. The aforementioned results also confirmed that the UCNPs-RB&ZnPc could be specifically localized in mitochondria of living cells (Fig. 6c) and reached high accumulation after 24 h. Based on this understanding we performed NIR-triggered PDT for cancer cells at the mitochondria enrichment time at 24 h. After another 3 h culture, the membrane potential (ΔΨm) of mitochondria was measured. As shown in Fig. 8a, no detectable loss in ΔΨm was found without light irradiation for cells treated with nanoplatforms. However, in the presence of 980 nm light, the NIR light triggered PDT caused great loss of mitochondrial membrane potential with green fluorescence as shown in Fig. 8b, which might result in heavy photodamage to mitochondrial membranes. Moreover, it is likely to suggest that the heavy dissipation of ΔΨm might cause the drop of intracellular ATP levels, the damage to mitochondria, and irreversible ensuring cell death. It is a feature of early apoptotic cells indicating a mitochondrial apoptosis pathway.

In addition, the PDT mediates cell death pathways include apoptosis, necrosis, and autophagy-associated cell death^30^,^31^. To detect the pathway of cell death induced by NIR light triggered PDT, the effect of UCNPs-RB&ZnPc on apoptotic death of A549 cells was assessed from annexin V-FITC and PI apoptosis experiment after 15 min PDT and further 3 h incubation, as shown in Fig. 8c. CLSM imaging with annexin V-FITC and PI staining can clearly distinguish early apoptosis from late apoptosis or necrosis. The cell membranes were stained in green and the nuclei without red, which is a character of apoptosis, indicating the occurrence of apoptosis at the early stage. The control group had very few cells staining, assuring that there was almost no phototoxicity without light irradiation (not shown). To further confirm the above results, the molecular mechanism of apoptosis related proteins of Bcl-2 family in NIR-triggered PDT was investegated by Western blotting, as sown in Fig. 8d. Compared with the blank control group and the non-irradiation group, we could find the increased levels in the ratio of proapoptotic proteins (Bak), together with a corresponding decline of antiapoptotic proteins (Bcl-2), demonstrating that the PDT activated mitochondria on A549 cells. Furthermore, upregulation of Bak can form multimers and cause cytochrome c release from mitochondria into the cytosol to promote cell apoptosis. As we can see that the protein expression levels of cytochrome c in the cytoplasm were significantly increased after the PDT, indicating the occurance of apoptosis. As we known, release of cytochrome c from the inter membrane spaces of the mitochondria into the cytosol is a key event in apoptosis via the mitochondria-mediated pathway. The above results revealed that the NIR- triggered PDT induced apoptosis followed a mitochondria-dependent mechanism.

### PDT *in vivo* and histological studies of tumors

In order to evaluate the efficiency of the optimized nanoplatform compared with those following single loading strategies, we carried out the PDT *in vivo*. Figure S3a presents contrast photographs of tumor-bearing mice before and after the treatment with different UCNPs-PSs. The subcutaneously injected Hepa1-6 cells in control groups (1) grew into ~700 mm^3^ solid tumors in two weeks after injection, while the tumor in treatment group (3, 5 and 7) was inhibited. The tumor volumes of all the control groups and treatment groups were given in Figure S3b. The tumor-bearing mice treated with saline and UCNPs-PS without light irradiation did not show any therapeutic effect. In contrast, the mice, which were treated with UCNPs-RB&ZnPc and 980 nm irradiation, exhibited the most significant decrease in tumor volume within two weeks and the relative tumor inhibitory ratio was calculated to be approximately 90% in comparison with group 1. It should be emphasized the lowest irradiation power used for the PDT based on UCNPs studies to date was 360 J/cm^2^ with the UCNPs-PS dosage of 50 mg/kg. However, the situation was greatly improved in our study that under the irradiation power of 225 J/cm^2^ and drug dosage of only 17 mg/kg, the tumor inhibition ratio can reach up to 90.1% by using the designed dual-loaded UCNPs-PS, evidencing the efficient energy transfer and thus improved singlet oxygen production.

Subsequently, the histological analysis on tumor, heart, liver, spleen, lung, and kidney was carried out in different treatment groups after 14 days of post-treatment as shown in Figure S4a. It can be clearly observed that the morphology, size and staining of the tumor cells treated with UCNPs-PS and without 980 nm irradiation are at variance, and mitotic figures are seen in most nuclei. However, markedly increased apoptotic and necrotic tumor cells were observed in all PDT treatment groups, especially in the group treated with dual-loaded sample. Histological analysis reveals no pathological changes in the heart, lung, kidney, liver or spleen. Hepatocytes in the liver samples were found normal. No pulmonary fibrosis was detected in the lung samples. The glomerulus structure in the kidney section was observed clearly. Necrosis was not found in any of the histological samples analyzed. These results clearly demonstrate the potential clinical applicability of UCNPs-RB&ZnPc as PDT agents. The detailed experimental protocols were represented in the supporting information.

## Conclusions

In summary, we have traced the subcellular dynamic distribution of the UCNPs-based nanophotosensitizers. It was clearly shown that the nanophotosensitizer of PAAm-UCNPs-RB&ZnPc was able to be endocytosed by the cellular uptake and transported to endomes/lysosomes and mitochondria in A549 cells. The corresponding PDT indicated that an effective PDT would take place when most of the nanophotosensitizers accumulated in mitochondria. Therefore the starting time of PDT, is crucial to reach a satisfactory therapeutic treatment, and the mechanism of the most efficient PDT is via the pathway of mitochondria-mediated apoptosis. Our work shall shed light on the biomedical application of the novel UCNPs-based nanophotosensitizers.

## Experimental

### Materials

YCl_3_·6H_2_O (99.9%), YbCl_3_·6H_2_O (99.9%), ErCl_3_·6H_2_O (99.9%), 1-octadecene (90%) (ODE), Oleic acid (OA), Y(CF_3_COO)_3_, Yb(CF_3_COO)_3_, CF_3_COONa Poly(allylamine) (PAAm), 1-ethyl-3-(3-dimethylaminopropyl) carbodiimide (EDC), N-hydroxy-succinimide (NHS), 1,3-diphenylisobenzofuran (DPBF), MTT, DAPI were purchased from Sigma Aldrich. Annexin V-FITC Apoptosis Detection Kit was obtained from keyGEN BioTECH. JC-1, Mito-tracker and Lyso-tracker were purchased from beyotime. mPEG-SC was purchased from XIAMEN SINOPEG BIOTECH CO., LTD. NaOH and NH_4_F were purchased from Beijing Chemical Works. All chemicals were used as received without further purification. Zinc chloride, Zinc (II)-phthalocyanine (ZnPc) and Rose Bengal-COOH was synthesized following our previously reported protocol^19^.

### Covalent conjugation of amino-functionalized core/shell UCNPs with RB and ZnPc photosensitizers

The obtained bare core of NaYF_4_:Yb,Er UCNP was synthesized following our previously reported route and the core/shell structure of UCNP was synthesized according to reported procedure with modification^32^. Briefly, 2 mL cyclohexane containing the as-prepared NaYF_4_:Yb,Er UCNP was mixed and dissolved in OA and OED. The temperature was gently raised to 65 °C to remove cyclohexane and subsequently heated to 300 °C for 5 min. After that, 1 mmol of NaYF_4_ inert shell in ODE was injected into the reaction mixture and ripened for 1 h. Finally, the solution was cooled down to room temperature and precipitated using ethanol twice and re-dissolved in cyclohexane for further use. Before the amino-functionalized UCNPs synthesis, the OA ligand on the core/shell UCNPs surface was removed. 2 mL cyclohexane containing 40 mg UCNPs added into 2 mL HCl solution (pH = 2). After 4 h stirring, the water layer was centrifugated (11500 rpm for 30 min) to obtain the ligand-free UCNPs. Finally 100 μL of PAAm was added to the ligand-free UCNPs dispersed in DMF solution overnight. After centrifugation twice, the as-prepared amino-UCNPs were dispersed in aqueous media for further conjugation. The RB-COOH, ZnPc-COOH and mPEG-SC was covalently conjugated to NH_2_-UCNPs by the condensation method of EDC and NHS separately with different amount of photosensitizer. Take RB-COOH for example, briefly, 1.2 mg RB-COOH dissolved in 5 mL DMF and 6 mg EDC was added to the above solution for 2 h. After that, 0.5 mL DMF containing functional UCNPs (10 mg/mL) and NHS (2.4 mg) was added to the above solution. The reaction mixture was vigorously stirred overnight. The mixture was then centrifuged at 11500 rpm for 30 min three times to spin down the nanoparticles. The conjugation of PEG-SC to the nanophotosensitizer was obtained by aminolysis reaction for enhancing biocompatibility. 4 mL of DMSO and ethanol mixture solution containing 1 mg PEG-SC and 10 mg UCNPs-RB@ ZnPc was stirred overnight. The mixture was then centrifuged at 11500 rpm for 30 min. After 3 times washing, the pellet was dissolved in PBS for further use. The obtained nanophotosensitizer were stable over half a year in water under ambient conditions.

### Singlet oxygen determination

The generation of singlet oxygen was detected by the DPBF probe. Briefly, 5 μL of a DPBF–ethanol solution (10 mM) was added to 2 mL of as-prepared UCNPs solution. Then the whole solution was irradiated using 980 nm laser light at the same power density (0.25 W/cm^2^), and the absorption of DPBF at 417 nm was measured every 2 minutes.

### Cellular viability, uptake and distribution assay and organelle guided PDT *in vitro*

For cell viability assay, A549 cells were cultured in RPMI-1640 (GIBCO), supplemented with 10% fetal bovine serum (FBS) in a 5% CO_2_ humidified circumstance at 37 °C. The A549 cells were seeded in 96-well plates (1 × 10^4^ cells per well). After cultivation for 24 h, 100 μL of UCNPs-PS with different concentrations were added into the wells and incubated for another 24 h. Then, MTT solution (20 μL, 5 mg mL^−1^) was added into each well. After incubation for 4 h at 37 °C, the MTT solution was replaced by 100 μL of DMSO in each well. The absorbance in each well was measured at 492 nm.

To evaluate the cellular uptake profiles, A549 cells were incubated in glass bottom cell culture dishes. After confluent growth for 24 h, the medium was removed and fresh medium containing UCNPs-RB&ZnPc (100 μg/mL) was added into the corresponding well and incubated at 37 °C for 2 h, after that, the cells were washed three times with PBS. Subsequently, cells were fixed with immunol staining fix solution for 15 min. Then the dishes were washed three times with PBS and were stained with DAPI. For further assessment of UCNPs-RB&ZnPc cellular uptake, the cellular internalization and subcellular distribution of nanoplatform was studied in lysosome (2 h, 8 h, 12 h and 24 h) and mitochondrial (2 h, 8 h, 12 h and 24 h). After then the dishes containing the corresponding stained cells were imaged with a modified confocal laser scanning microscope under excitation of 980 nm laser light (CLSM, Nikon microscope with a CCD camera). Guided by the distribution and enhancing accumulation of nanoplatform in the subcellular organelles as time changed. The PDT effect of UCNPs-RB&ZnPc at different time was carried out against cancer cells with 980 nm light for 15 min (3 min break after 5 min irradiation at 0.25 W/cm^2^). In order to evaluate the PDT effect accurately, we strictly control the amount of photosensitizer loading and the single-loading UCNPs-RB and UCNPs-ZnPc was assessed respectively for control.

### Mitochondrial membrane potential and annexin V-FITC/PI detection for apoptosis by CLSM imaging

Since mitochondria membrane potential (MMP, ΔΨm) is an early event in apoptosis, JC-1 staining was used to evaluate the alterations in ΔΨm. Adhered cells were seeded onto 33 mm glass bottom cell culture dishes for 24 h and treated in the same way as for UCNPs-RB&ZnPc uptake. Subsequently, after PDT treatment and a further 3 h, the treated cells were washed three times with PBS and incubated in pre-warmed complete medium containing 1.0 μg/mL mitochondrial JC-1 dye for 20 min at 37 °C. Stained cells were washed and incubated in fresh medium. Similarly, the apoptosis in PDT treated cells was detected by using annexin V-FITC/PI apoptosis detection kit and obtained by confocal imaging.

### Western Blotting Analysis

SDS-PAGE and immunoblotting were performed according to standard procedures to assay the expression levels of apoptosis-associated proteins including cytochrome C, Bak and Bcl-2. Briefly, after 3 h incubation flollowing NIR PDT, the corresponding proteins as above were extracted and separated by SDS–PAGE 12% Tris-glycine gels. After that the proteins were electrophoretically transferred to PVDF membranes for 1.5 h at 100 V using a Mini Trans-Blot electrophoretic transfer cell. The membranes were blocked with 5% non-fat milk power (w/v) in PBST (1x PBS and 0.05% Tween 20) for 1 h at room temperature. The specific primary antibodies were used to probe the protein levels of the different desired molecules overnight at 4 °C, followed by the appropriate peroxidase-conjugated secondary antibodies for 40 min at room temperature. The blots were visualized by Western blotting detection reagents containing DL-p-Chloroamphetamine hydrochloride (PCA) and luminol according to the recommended procedure.

## Additional Information

**How to cite this article**: Chang, Y. *et al*. Precise Photodynamic Therapy of Cancer via Subcellular Dynamic Tracing of Dual-loaded Upconversion Nanophotosensitizers. *Sci. Rep.* **7**, 45633; doi: 10.1038/srep45633 (2017).

**Publisher's note:** Springer Nature remains neutral with regard to jurisdictional claims in published maps and institutional affiliations.

## Supplementary Material

Supplementary Information

## Figures and Tables

**Figure 1:**
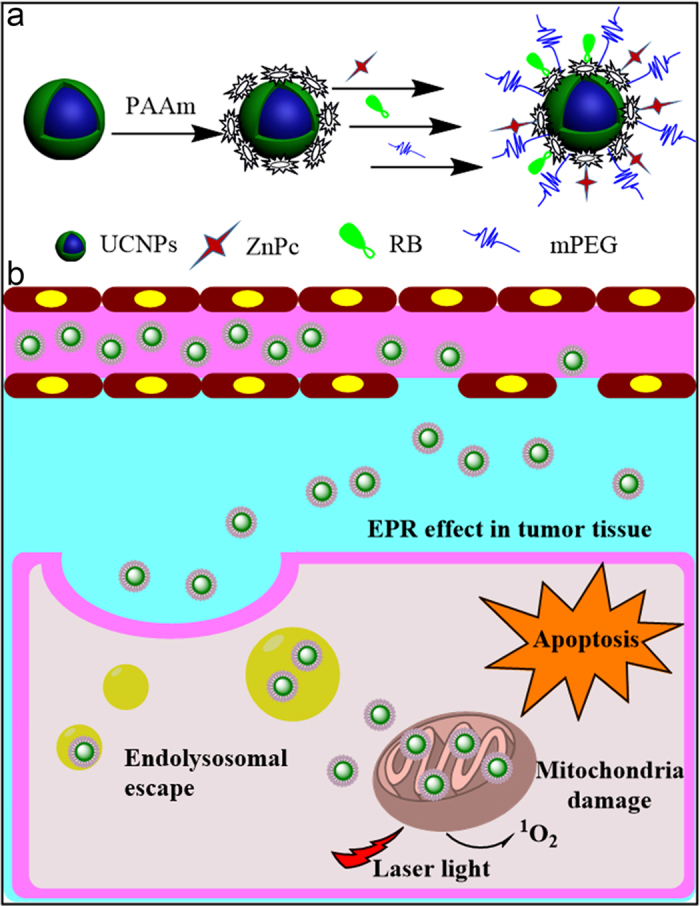
(**a**) Construction of core/shell co-loading UCNPs-RB&ZnPc nanoplatform (**b**) the intracellular nanoplatform-organelle interactions and mechanism of PDT.
