# Supplementary material for: Precise Photodynamic Therapy of Cancer via Subcellular Dynamic Tracing of Dual-loaded Upconversion Nanophotosensitizers
Source: Sci Rep. 2017 Mar 31;7:45633. doi: 10.1038/srep45633 (PMC5374495; doi:10.1038/srep45633)
Supplement: Supplementary Information [file srep45633-s1.pdf]

Supporting information

Precise Photodynamic Therapy of Cancer via Subcellular Dynamic Tracing of Dual-loaded Upconversion Nanophotosensitizers

Yulei Chang[1], Xiaodan Li[1,3], Li zhang[1,3], Lu Xia[1], Xiaomin Liu[1], Cuixia Li[1], Youlin Zhang[1], Langping Tu[1,2], Bin Xue[1,2], Huiying Zhao[3], Hong Zhang[2*] & Xianggui Kong[1*]*

[1] State Key Laboratory of Luminescence and Applications. Changchun Institute of Optics, Fine Mechanics and Physics, Chinese Academy of Sciences. 130033, Changchun, Jilin, China.

[2] Van't Hoff Institute for Molecular Sciences. University of Amsterdam, Science Park 904, 1098 XH Amsterdam, The Netherlands.

[3] The First Hospital of Jilin University, Changchun 130021, China.

E-mail: xmliu@ciomp.ac.cn (Liu); h.zhang@uva.nl (Zhang); xgkong14@ciomp.ac.cn (Kong)

1. Experimental Procedures

1.1 **Assessing the integrity of the lysosomal membrane**

The integrity of the lysosomal membrane was assessed qualitatively by confocal microscopes. A549 Cells were seeded onto 35 mm dishes for 24 h and rinsed twice with PBS. Afterwards, cells were incubated with 5 μg/mL of AO for 15 min before rinsing three times with PBS. Then, lysosomal membrane permeation of A549 cells was analyzed after 6 h continuous incubation by as-prepared nanoplatfrom. Finally, the cells were observed with confocal microscope and samples were excited at 488 nm. Emission was detected at green and red channel, respectively.

1.2 In vivo **efficacy**

Animals received care in accordance with the Guidance Suggestions for the Care and Use of Laboratory Animals. All the animals received care complied with the guidelines in the Guide for the Care and Use of Laboratory Animals, and the procedures of animal experiments were approved by the Institutional Ethical Committee of Animal Experimentation of Jilin University. The methods were carried out strictly in accordance with governmental and international guidelines on animal experimentation. All efforts were made to minimize the usage amount of animals and the suffering during experiments according to the request of Biosafety and Animal Ethics.

All the animal studies were carried out in compliance with the animal management protocols. Tumor-bearing mice were produced by the subcutaneous injection 100 μL of Hepa1-6 cells in female C57/6J mice (about 20 g, Jilin University Animal Center). The mice were randomly assigned to eight groups (n=6 per group) for various treatments (received intratumoral direct injection) as the tumor grew to about 100 mm^3, as follows: (1) group 1: the saline (the control group); (2) group 2: the UCNPs-RB without 980nm irradiation; (3) group 3: the UCNPs-RB with NIR laser irradiation; (4) group 4: the UCNPs-ZnPc without 980 nm irradiation; (5) group 5: the UCNPs-ZnPc with NIR laser irradiation; (6) group 6: the UCNPs-RB&ZnPc without 980 nm irradiation; (7) group 7: the UCNPs-RB&ZnPc with NIR laser irradiation. The NIR laser irradiation groups were irradiated with 980 nm laser (0.25 W/cm^2) for 15 min and. All the mice were fed normally and monitored every three days to evaluate the treatment effect. The method for evaluating therapy efficacy was according to a previous study.[1-2]

1.3 Histology analysis *ex vivo*

Histology examination was performed at the 14th day after treatment. The organs (heart, liver, spleen, lung and kidney) and tumor tissues of the mice from each group were harvested from the mice, fixed in 10% neutral buffered formalin and processed into paraffin. The sliced organs and tumor tissues were stained with Hematoxylin and Eosin (H&E) and examined by a Nikon CS2 microscope.

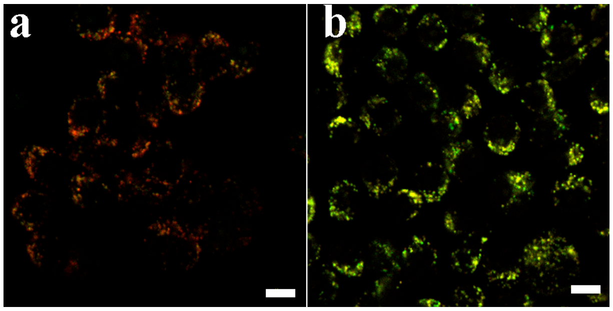

Figure S1. The integrity of the lysosomal membrane (AO staining) in A549 (a) without UCNPs-PS as control, (b) with 100 μg/mL of UCNPs-PS by confocal microscopy after incubation 6 h. Scale bar, 10 μm.

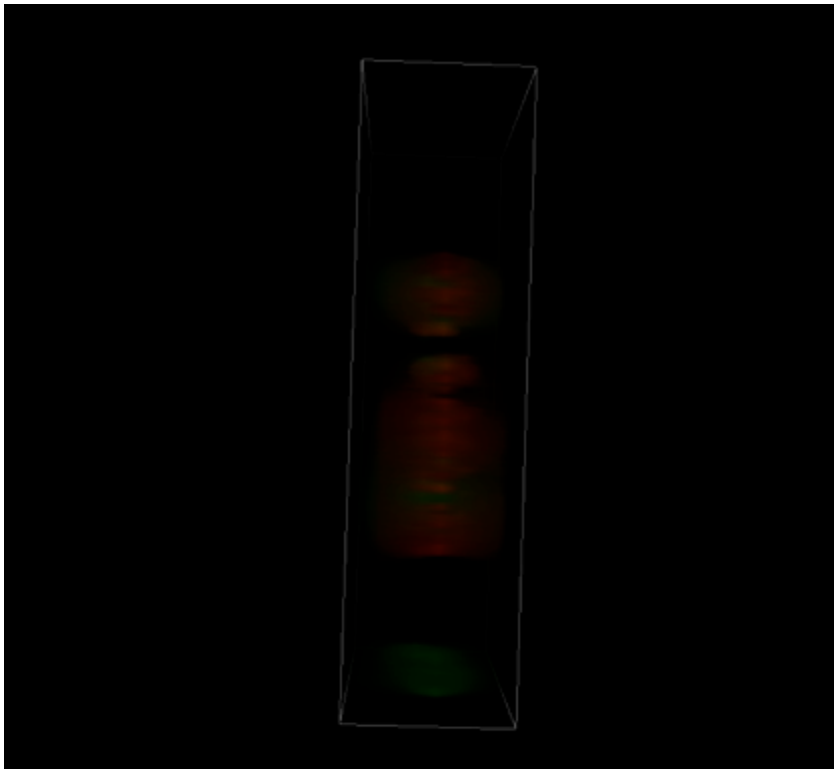

Figure S2. Z-scanning micrographs of A549 cells after 24 h incubation of UCNP-PS, which was provided by the CW laser at 980 nm, the red UCL emission of UCNPs and the green emission from Mito-tracker labeled in mitochondria.

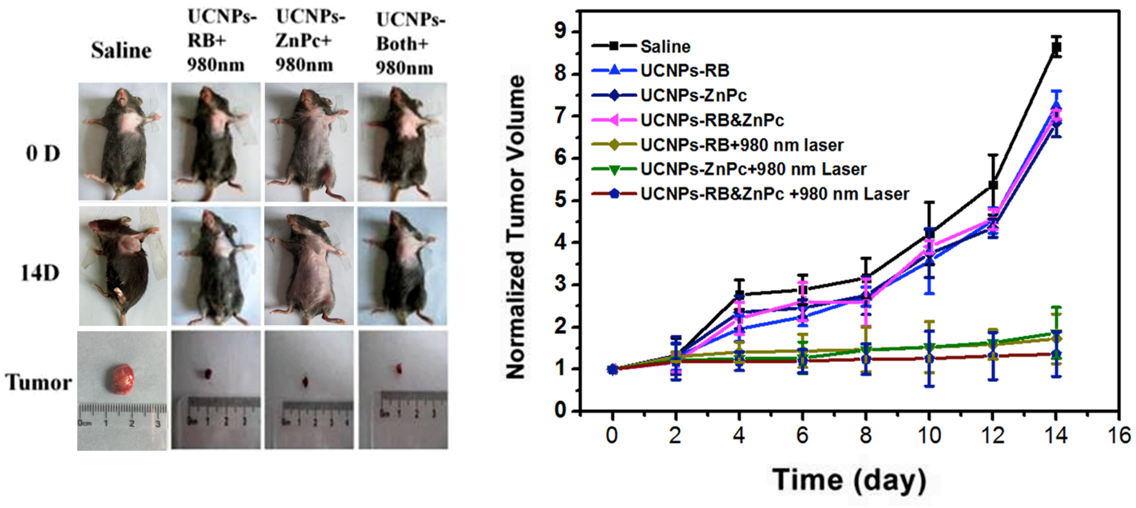

Figure S3 (a) Representative photos of mice and liver tumor before and after various treatments, and photos of tumor tissue were obtained after 14 days. (b) The growth of Hepa1-6 tumor in different groups after treatment.

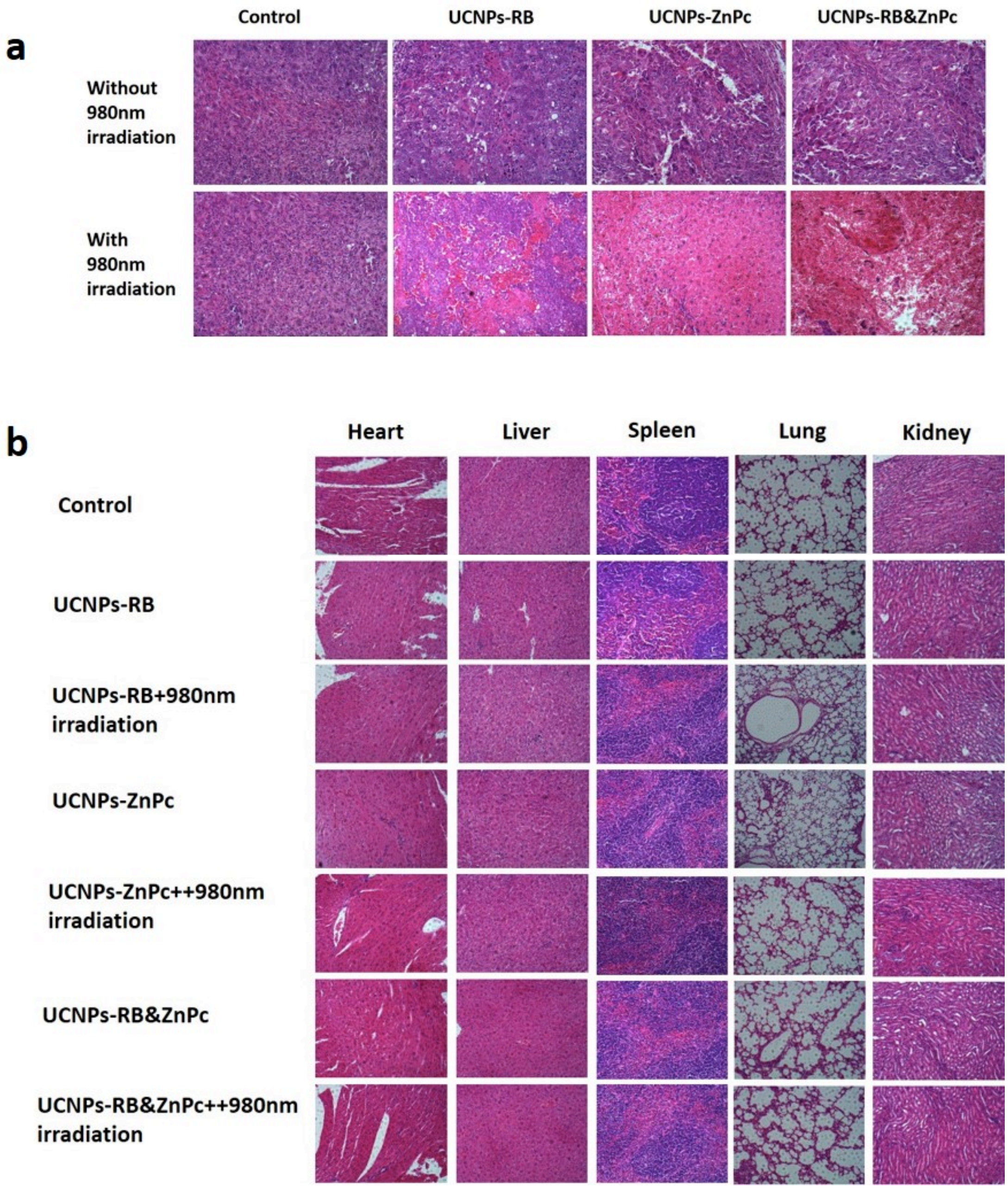

Figure S4. Representative histological H&E stained organ tissues images of (a) tumor, (b) heart, liver, spleen, lung, kidney collected from different groups.

Figure S5. Uncropped Western blots related to main Figure 8.

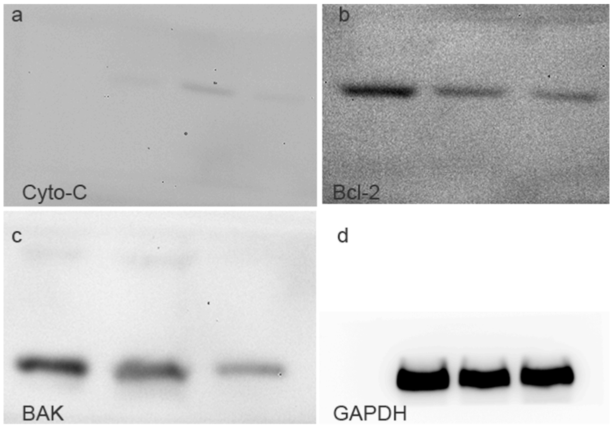

References for Supporting Information

1. Xia, L.; Kong, X. G.; Liu, X. M.; Tu, L. P.; Zhang, Y. L.; Chang, Y. L.; Liu, K.; Shen, D. Z.; Zhao, H. Y.; Zhang, H., An upconversion nanoparticle - Zinc phthalocyanine based nanophotosensitizer for photodynamic therapy. *Biomaterials* **2014,** *35* (13), 4146-4156.

2. Chang, Y.; Li, X.; Kong, X.; Li, Y.; Liu, X.; Zhang, Y.; Tu, L.; Xue, B.; Wu, F.; Cao, D.; Zhao, H.; Zhang, H., A highly effective in vivo photothermal nanoplatform with dual imaging-guided therapy of cancer based on the charge reversal complex of dye and iron oxide. *J. Mater. Chem. B* **2015**.
